# Supplementary figures and images for: Job postings in the substance use disorder treatment related sector during the first five years of Medicaid expansion
Source: PLoS One. 2020 Jan 30;15(1):e0228394. doi: 10.1371/journal.pone.0228394 (PMC6992002; doi:10.1371/journal.pone.0228394)

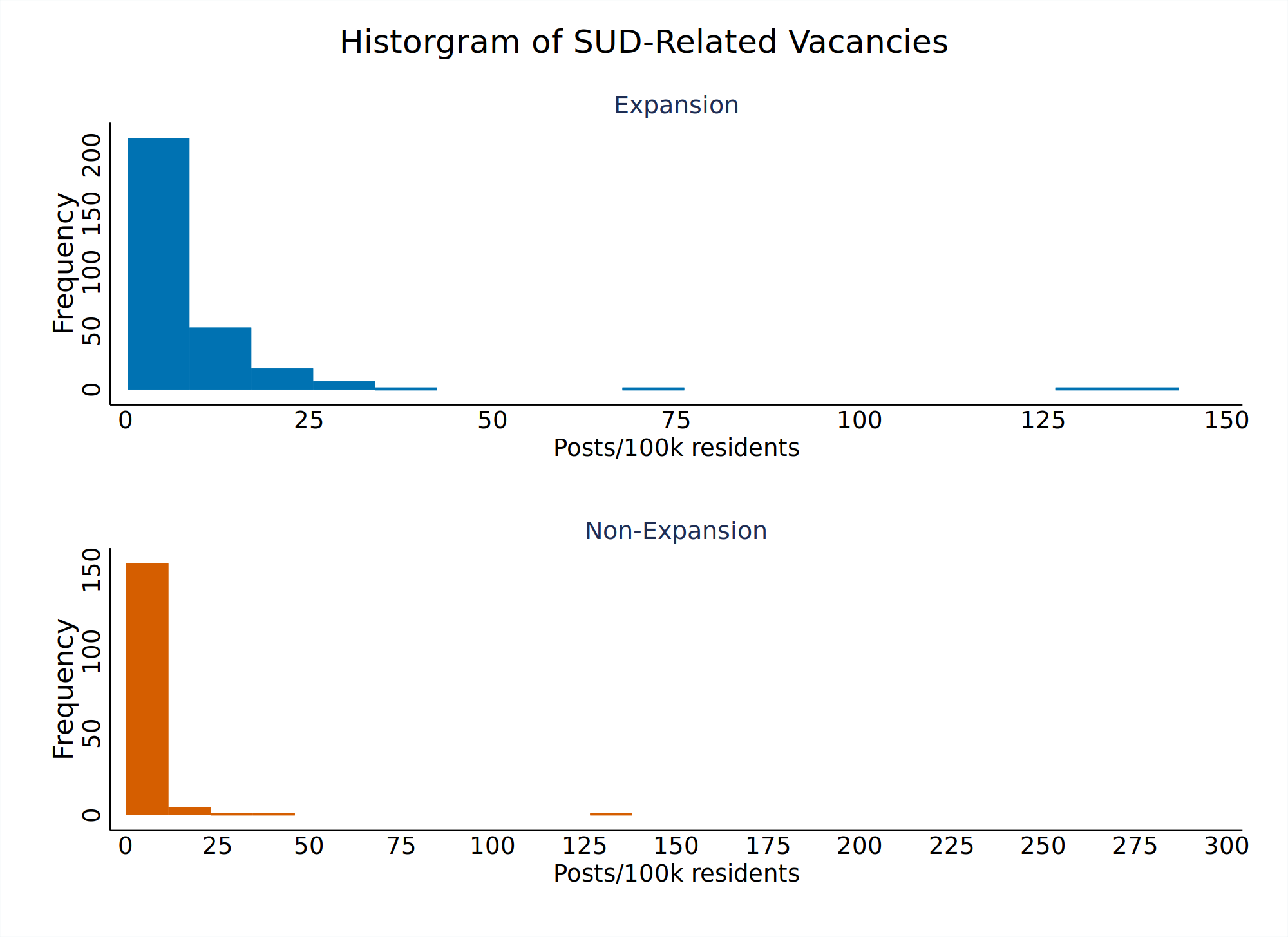

Supplement: S1 Fig — Authors’ calculations based on BGT, 2010-18. (TIF) [file pone.0228394.s001.tif]

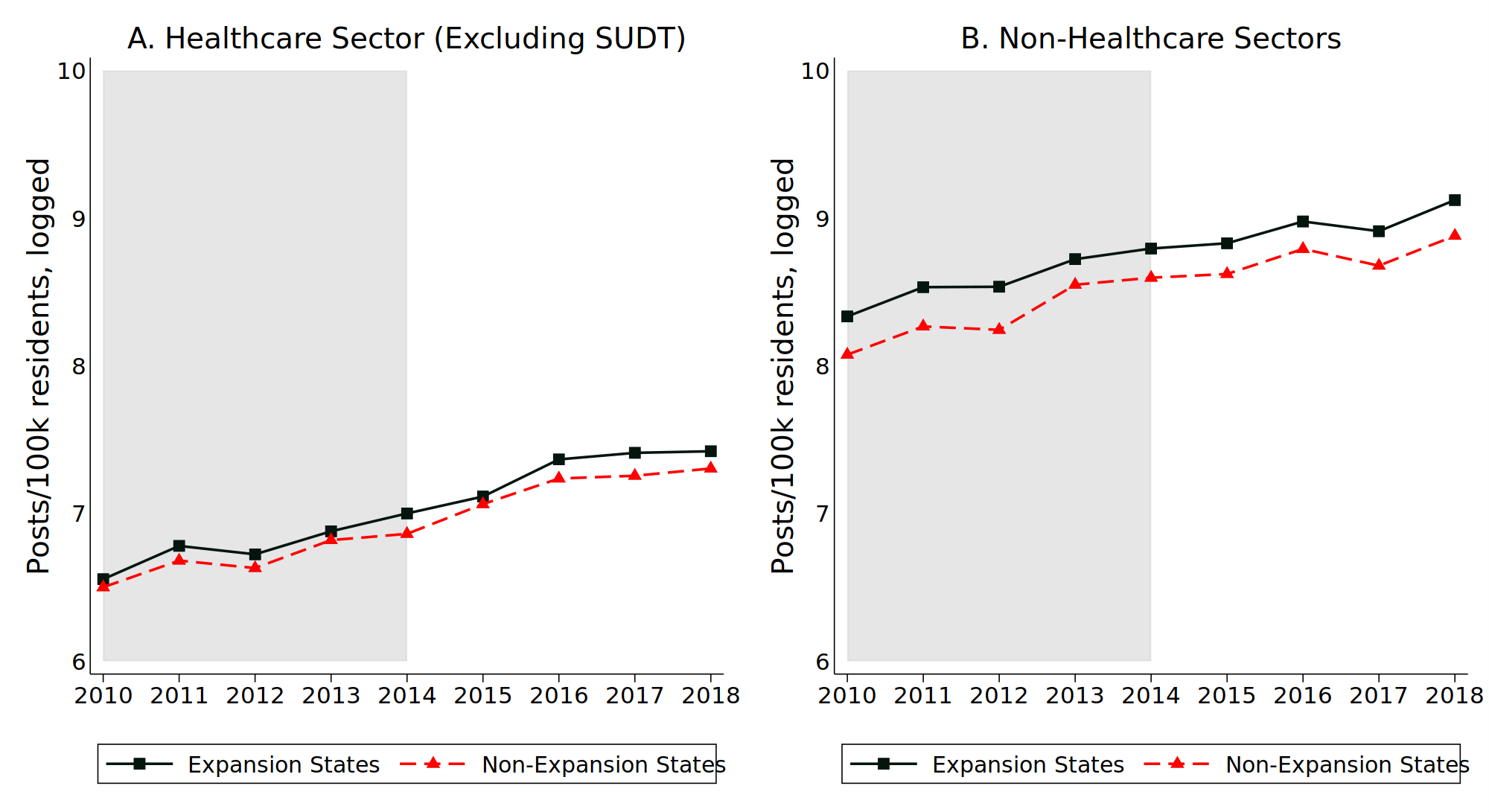

Supplement: S2 Fig — Authors’ calculations based on NAICS-state data from BGT, 2010-18. In particular, we used the NAICS-state data to compare means of job postings for Expansion States and Non-Expansion States. Estimates were adjusted by state populations. ME and late expansion states (AK, IN, LA, NH, MI, MT, and PA) were excluded from the calculations. (TIF) [file pone.0228394.s002.tif]

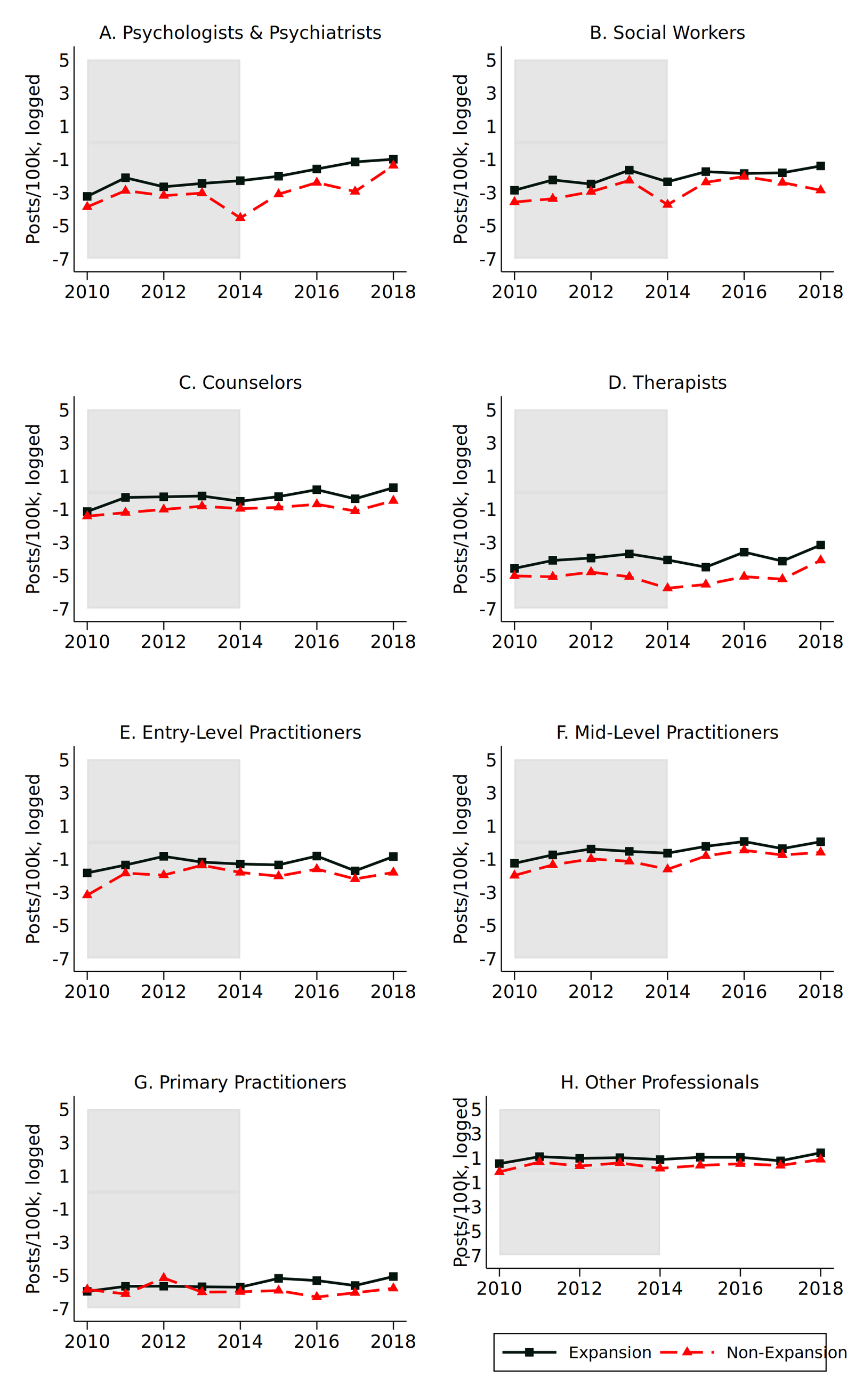

Supplement: S3 Fig — Authors’ calculations based on NAICS-state data from Burning Glass, 2010-18. Estimates were adjusted by state populations. Late expansion states (AK, IN, LA, NH, MI, MT, and PA) were excluded from the calculations. (TIF) [file pone.0228394.s003.tif]

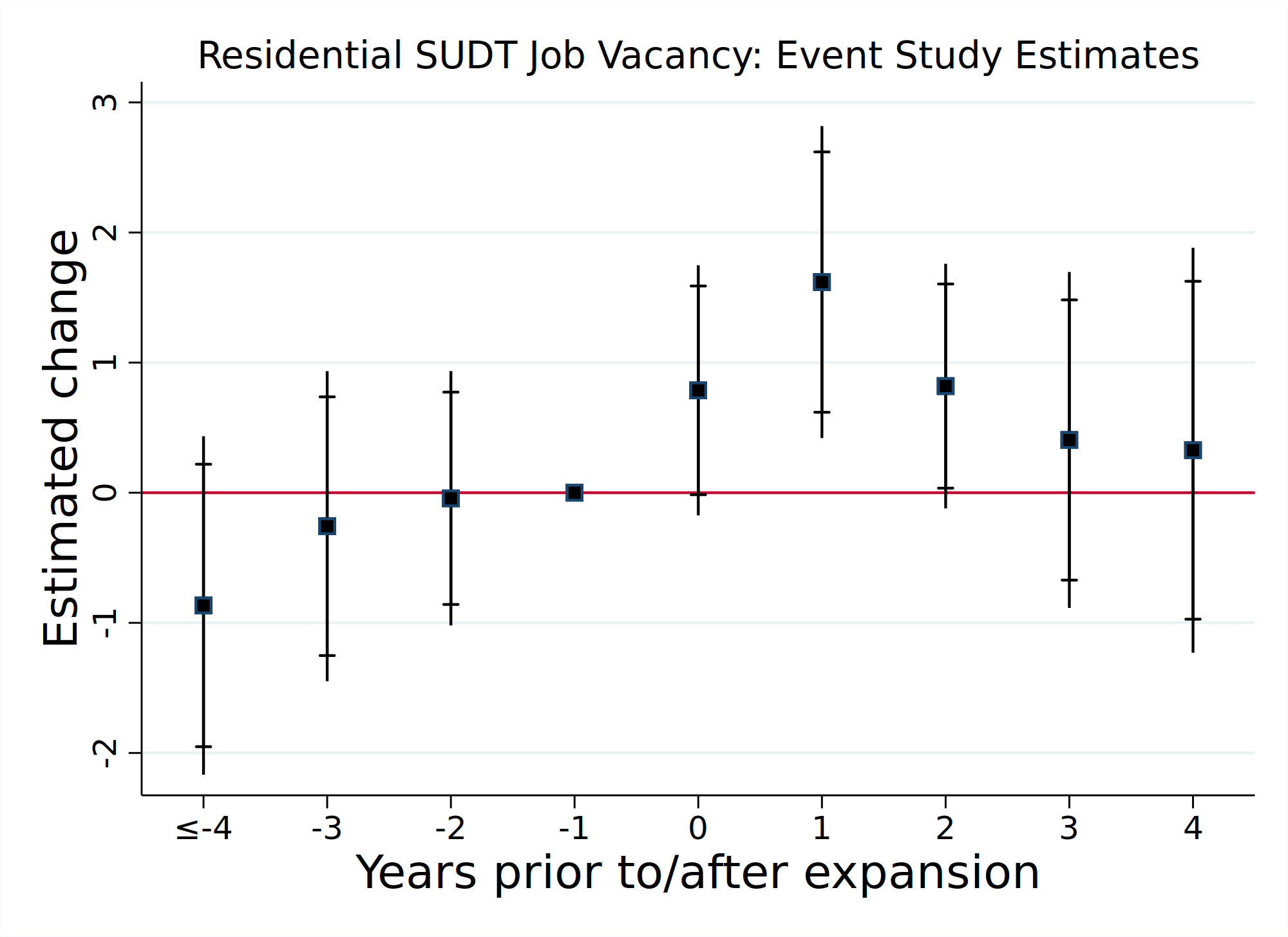

Supplement: S4 Fig — The dependent variable is the number of job postings in residential SUDT centers per 100,000 state residents, which takes a logged form. (TIF) [file pone.0228394.s004.tif]

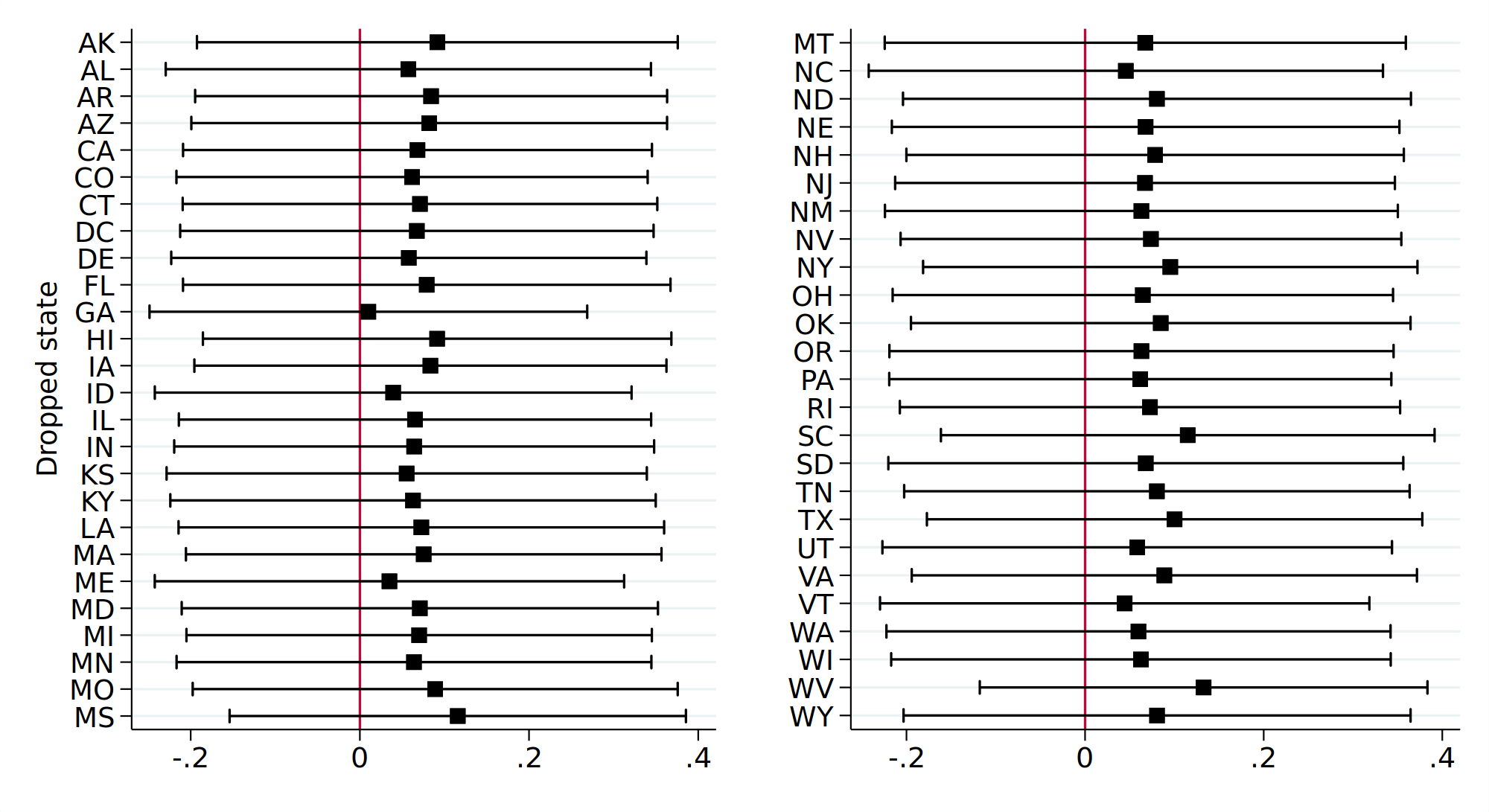

Supplement: S5 Fig — This figure shows the DD Estimates and their 95% CIs for Impact of Medicaid Expansion on the number of job postings per 100,000 state residents. (TIF) [file pone.0228394.s005.tif]
