# Supplementary material for: Job postings in the substance use disorder treatment related sector during the first five years of Medicaid expansion
Source: PLoS One. 2020 Jan 30;15(1):e0228394. doi: 10.1371/journal.pone.0228394 (PMC6992002; doi:10.1371/journal.pone.0228394)
Supplement: S1 Table — Column 1: the dependent variable is the number of job postings per 100,000 state residents, which takes a logged form. A small amount (0.001) was added to this outcome in order to remove zeros in these analyses. Column 2: the dependent variable is the count of job postings. Column 3: the dependent variable is the number of job postings per 10,000,000 state residents, rounded to a count variable. * p<0.1 ** p<0.05 *** p<0.01. (PDF) [file pone.0228394.s006.pdf]

S1 Table DD Estimates for Impact of Medicaid Expansion on Job Postings of SUDT-Related Industries - Robustness Check with Different Methods Column 1: the dependent variable is the number of job postings per 100,000 state residents, which takes a logged form. A small amount (0.001) was added to this outcome in order to remove zeros in these analyses. Column 2: the dependent variable is the count of job postings. Column 3: the dependent variable is the number of job postings per 10,000,000 state residents, rounded to a count variable. * p<0.1 ** p<0.05 *** p<0.01.

	Model 1<br>OLS	Model 2<br>Negative Binomial	Model 3<br>Negative Binomial
Expansion×Post-2014	0.072	0.18	0.14
	(0.14)	(0.15)	(0.14)
Unemployment rates, %	-0.018	-0.0060	-0.023
	(0.044)	(0.052)	(0.052)
Median income, logged	0.25	0.54	0.37
	(1.57)	(1.54)	(1.54)
Opioid prescribing rates	-0.0029	-0.0036	-0.0037
	(0.011)	(0.014)	(0.014)
Drug poisoning death rates	-0.0085	-0.0062	-0.0084
	(0.0083)	(0.0077)	(0.0079)
Dep. Variable Mean	1.42	311.81	698.22
Dep. Variable SD	0.95	400.73	1261.79
Observations (state-year)	459	459	459
