# Supplementary material for: Job postings in the substance use disorder treatment related sector during the first five years of Medicaid expansion
Source: PLoS One. 2020 Jan 30;15(1):e0228394. doi: 10.1371/journal.pone.0228394 (PMC6992002; doi:10.1371/journal.pone.0228394)
Supplement: S2 Table — Column 1: the dependent variable is the number of job postings per 100,000 state residents, which takes a logged form. A small amount (0.001) was added to this outcome in order to remain zeros in these analyses. Column 2: the dependent variable is the count of job postings. Column 3: the dependent variable is the number of job postings per 10,000,000 state residents, rounded to a count variable. * p<0.1 ** p<0.05 *** p<0.01. (PDF) [file pone.0228394.s007.pdf]

S2 Table DD Estimates for Impact of Medicaid Expansion on Job Postings of SUDT-Related Industries - Robustness Check with an Alternative Policy Coding and Different Specifications Column 1: the dependent variable is the number of job postings per 100,000 state residents, which takes a logged form. A small amount (0.001) was added to this outcome in order to remain zeros in these analyses. Column 2: the dependent variable is the count of job postings. Column 3: the dependent variable is the number of job postings per 10,000,000 state residents, rounded to a count variable. * p<0.1 ** p<0.05 *** p<0.01.

	Model 1<br>Baseline	Model 2<br>No early adopters	Model 3<br>Specification 1	Model 4<br>Specification 2
Expansion×Post-2014	0.072	0.050	0.048	
	(0.14)	(0.14)	(0.14)	
Lagged [Expansion×Post-2014]				0.024
				(0.15)
Unemployment rates, %	-0.018	-0.016	-0.021	-0.018
	(0.044)	(0.043)	(0.039)	(0.044)
Median income, logged	0.25	-0.44	0.22	0.22
	(1.57)	(1.81)	(1.59)	(1.57)
Opioid prescribing rates	-0.0029	-0.0023		-0.0030
	(0.011)	(0.012)		(0.011)
Drug poisoning death rates	-0.0085	-0.011		-0.0078
	(0.0083)	(0.010)		(0.0084)
Dep. Variable Mean	1.42	1.30	1.42	1.42
Dep. Variable SD	0.95	0.87	0.95	0.95
Observations (state-year)	459	414	459	459
