# Supplementary material for: Job postings in the substance use disorder treatment related sector during the first five years of Medicaid expansion
Source: PLoS One. 2020 Jan 30;15(1):e0228394. doi: 10.1371/journal.pone.0228394 (PMC6992002; doi:10.1371/journal.pone.0228394)
Supplement: Attachment — Submitted filename: Response to Reviewers.pdf [file pone.0228394.s009.pdf]

INDIANA UNIVERSITY
PAUL H. O'NEILL SCHOOL OF PUBLIC AND ENVIRONMENTAL AFFAIRS

Response to Reviewers

Dear Editor:

We are grateful for the extremely helpful review of our manuscript on *Hiring in the substance use disorder treatment related sector during the first years of Medicaid expansion*.

We submit here a revised version of our article, which incorporates the changes recommended by the reviewers. We also include a point-by-point response to each of their comments.

Thank you again for your continued consideration of our work. We hope this revision is sufficiently responsive to the first round of feedback to merit publication, but we stand ready to make whatever adjustments you deem necessary.

<u>In response to comments from Reviewer 1:</u>

1. One of my major questions for the authors concerns the Burning Glass Technologies data. Does the data measure the number of workers firms are seeking to hire or the number of job postings? For example, if I run a SUDT facility and I place one job advertisement seeking to hire three counselors, will that show up as ONE in the BGT data or THREE?

<u>Response:</u>

Thank you for raising this important question. The short answer is that BGT data can only capture whether there is a hiring attempt, not how many may be hired through one ad. The longer answer is that the BGT data measures of Online Job Posting data capture the same concept as and correlates closely with the Bureau of Labor Statistics measures for job openings by industry [27]. The employers may advertise the same job opening on multiple sites and on different dates, which is a further hurdle for accurately measuring hiring activity through online postings. BGT applies "deduplication" procedures, removing postings with the same job title, employer, and location that recur within a 2-month window [29] as those would simply be repeated postings of the same hiring attempt activity. Thus, the number of job postings correspond solely to the job availability (vacancy) and not to how many employees a company is hiring. We now explain this in more clear detail on page 5.

2. I also wondered if the authors' results change if they do not control for the opioid prescribing rates and drug poisoning mortality rates. It seems that both of these rates are plausibly affected by

1315 E. Tenth Street Bloomington, IN 47405-1701 fax (812) 855-7802

the Medicaid expansions and also by employment in the SUDT areas, so including them in the models might lead to “overcontrolling” bias.

Response:

We agree that it is valuable to re-estimate our models without controlling for factors that may themselves be affected by Medicaid expansion, as inclusion of those variables might lead to Medicaid expansion not showing its full effects. We have now added a sensitivity analysis which does not control for the opioid prescribing rates and mortality rates. We obtain results in this sensitivity analysis which are similar to the baseline results (Table S2), thus it does not appear that the controls were masking larger effects. We have added the following text on page 11:

Subsection: Covariables:

“To address some concern regarding potential ``overcontrolling bias'' we do not control for the opioid prescribing rates and mortality rates in a sensitivity analysis as these factors may be associated with Medicaid expansion.”

Subsection: Sensitivity Analyses

“In a sensitivity analysis for the main results (Model 3, Table S2), we do not control for the opioid prescribing rates and mortality rates as these factors may be associated with Medicaid expansion. The results are similar to those of the baseline regression.”

3. I was intrigued by the occupation-specific results, and I have some thoughts on these models. First, the authors state on pg. 9 that, “although (perhaps unsuprisingly given the number of different specifications we run), there are some coefficients that are statistically significant for specific occupations.” Can the authors adjust their p-values for multiple-hypothesis tests? That might be an easy way to confront whether the large number of hypotheses they test are driving some to be statistically significant by chance.
The authors talk some in the Discussion (on pg. 10) about the different levels of employment in the SUDT industry. It might be nice to have a slightly longer discussion about the potential mechanisms behind why hiring changed in some SUDT occupations but not in others. Finally, I wasn’t sure what to make of the occupation-specific results in Figure 4 compared to the all professionals results. The estimate for every single occupation (in both the extensive and overall results) seems to be higher than the all professions results. I would have thought that the all professions results would have been closer to the average of the different occupations. Do the authors have an idea on what’s going on? I apologize if I am missing something obvious.

Response:

First, thank you for raising the concern about testing multiple hypotheses. We addressed your comment by using a Bonferroni correction to adjust p-values to reduce the chance that the results include a false positive. However, with our updated results, most of the tests are not significant at the conventional level of significance.

Second, our updated results show no detectable effects of Medicaid on the number of hiring attempts on aggregate or for any specific occupation.

Third, you raised concern about the “adding up” of the estimates on occupation-specific results. Our earlier results on the number of postings for every single occupation actually fluctuated around the estimate on the total posting of all professionals (Figure 4 –B, in the previous version), but your observation about the estimates on the binary outcomes (Figure 3 – B in the previous version) were different compared to the estimations on numerical aggregate outcomes.

In the revised version, we manually cleaned the data, with input by BGT, to arrive at the appropriate industry codes to use in our analysis (namely naics4 level for SUDT hospitals and naics6 level for Outpatient and Residential SUDT, which lead to the inclusion of a larger number of job postings related to hospital SUDT facilities that are unclassified on naics6 level in BGT data. Due to this improved data set construction, we observed that every state had at least one job posting every year. Therefore, we do not now estimate the effects of Medicaid expansion on the extensive margin of hiring attempts (whether a state had a job posting in a certain year).

4. I’m confused why the authors didn’t run a Poisson QML model or Negative Binomial model with state and year fixed effects if their data is right-skewed. My understanding is that both of these models allow two way fixed effects, and including robust standard errors in a Poisson model will account for overdispersion.

Response:

Thank you for suggesting this. Our preferred estimation approach is the ordinary least squares (OLS) and log transformation of job postings per residents (our main outcome variable) as this approach is one of the most widely used and flexible estimation methods. Unlike the OLS models, the Negative Binomial model is appropriate to fit count outcomes, and we have now provided an additional table (S1) to compare the estimates using OLS and Negative Binomial models using count data versions of the dependent variable. As you will see, the results are similar.

We have added the following text on page 11:

Newly added Subsection: Sensitivity Analyses:

“We tested the sensitivity in our estimation method through two negative binomial models. The count outcome variable in the first negative binomial is the count of job postings by employers hiring in the SUDT workforce in a state (Model 2, Table S1). We additionally controlled for state populations in Model 2. The count outcome variable in the second negative binomial model is the count of job postings per 10,000,000 residents which was rounded to an integer in this count model (Model 3, Table S1). We used a large denominator in order to increase the accuracy of this rounding exercise. In these count models, we also controlled for state and year fixed effects. The DD estimates using negative binomial models are similar to those of the baseline model: the coefficients are positive and statistically insignificant.”

5. Are there any treatment states that are driving the authors' results? Can the authors run a “leave out” analysis where the drop one treatment state at a time to see if any states are extremely influential in the aggregate results.

Response

This is a great suggestion. We provided a “leave out” analysis in the revised version. We did not observe any particular state (either a treatment state or control sate) to be extremely influential in the results.

We have now added the following text to discuss this leave-one-out analysis on page 11.

“We tested whether our main results are driven by a particular state by using a “leave-one-out” test. We conducted this test by leaving out one of 50 states and Washington D.C. in the regression process and plotted the DD estimates in Figure S3. The results imply that there is no particular state (either a treatment state or control sate) that has an extreme influence on the baseline results.”

In response to comments from Reviewer 3:

Reviewer #2: This study examines the effect of public health insurance expansions on substance use disorder treatment (SUDT) employer labor demand proxied by job postings in SUDT facilities. The analysis uses a standard DD approach for the 2010-2018 analysis period to explore the impact of Medicaid expansions on SUDT job postings in aggregate or separated by occupation. The results suggest no statistically significant effect of the public expansions on the number of SUDT job ads as a whole, but some effects when looking at occupations separately.

I have the following comments/suggestions:

1. The title is a bit misleading. Maybe the authors can think about changing the word “hiring” as the study explores job postings that might proxy employer demand, not hiring.

Response

Thank you for pointing this out. Indeed, our study explores a job posting landscape which indicates industry demands and not actual hiring. We have updated the title to "**Job postings** in the substance use disorder treatment related sector during the first five years of Medicaid expansion".

2. While the focus of the analysis is job postings in SUDT agencies, the introduction is focused on opioid use disorders, their burden, and treatment. While opioid use is a growing concern, especially relative to other substances, I feel that the introduction should discuss SUDs in general. After reading the introduction, I was expecting an analysis focused on OTPs and OUDs.

Response:

Thank you for this suggestion. We have revised the introduction so now focuses on SUDs. It now reads:
"Worldwide, the direct burden of illicit drug dependence increased to 20 million disability-adjusted life years in 2010 [1]. Examples of these illicit drugs are opioids, cocaine, amphetamines, and cannabis which have been prohibited under international drug control treaties. Opioids have substantially contributed to this increased burden of substance use disorders (SUDs) due to its link to premature mortality, high disability, and the relatively large population with addiction. In the US, mental health and SUD together became the leading cause of disease burden in 2015, while nearly 3% of Americans aged 12 years or older reported SUDs in the same year [2].

The most effective SUD treatment (SUDT) is a combination of long-acting medications (usually methadone or buprenorphine) administered as part of a cognitive behavioral approach (such as counseling, family therapy, and peer support programs) [3]. The National Survey of Substance Abuse Treatment Services (NSSATS) reports that in 2017 there were 13,857 treatment facilities in the US with over 1,356,015 clients enrolled, representing only a 19% increase in total clients served since 2007 [4]. Opioid treatment programs (OTPs) are examples of these facilities where patients can obtain methadone. Despite increasing demand and perpetual waitlists for treatment, the supply of OTPs has remained low and constant over time, with around 1,500 approved programs in 2017 compared to 1,166 OTP programs reported in 2010 [5]. Alternatively, SUD patients can receive buprenorphine maintenance therapy from office-based providers (physicians, nurses practitioners and physicians' assistants) approved to prescribe buprenorphine [3]. Lack of buprenorphine-waivered providers is prevalent; in 2016 no buprenorphine waivered providers were found in 47% of all US counties, and in 72% of rural counties [6]. Persistent workforce barriers, leading to treatment underutilization, include insufficient education and training, burdensome regulatory procedures, lack

of ability to refer patients for mental health and substance abuse counseling, burdensome reimbursement barriers, and provider stigma [5]."

3. The authors use a Medicaid expansion coding scheme from the KFF. Several studies, however, follow a different coding scheme (see Miller, Wherry). According to this scheme, 5 states (DE, DC, MA, NY, VT) are considered to have adopted the expansions pre-2014. I would conduct a sensitivity test where these 5 'early adopters' are excluded from the analysis sample to check if the results change.

Response:

In the revised version, we have provided a sensitivity test where we dropped these 5 early adopters from the analysis. We retained results similar to baseline in this robustness check. We have now added the following text to discuss this alternative coding scheme on page 3.

In subsection: Medicaid

"Washington D.C., Delaware, Massachusetts, New York, and Vermont are consider early adopters of Medicaid expansion as they already provided similar coverage to low income adults. Similar to prior work, we included these early adopters in the main analysis [19]. However, we also conducted a sensitivity analysis on our policy coding scheme which excluded these five states and got results similar to baseline [20]."

Newly added Subsection: Sensitivity Analyses

"We tested whether our main results are driven by a particular state using a leave-one-out test. We conducted this test by leaving out one of 50 states and Washington D.C. in the regression process and plotted the DD estimates in Figure S3. The results imply that there is no particular state (either a treatment state or control sate) that that has an extreme influence on the baseline results."

4. While the authors explore heterogeneity by occupation and they point out that these outcomes' distribution demonstrates a strong positive skewness, I was wondering whether a better disaggregation would be by treatment agency setting. The authors note that job postings data contains the SUDT facility setting (hospital, residential, outpatient). Heterogeneity by treatment setting might result if Medicaid plans cover more generously outpatient treatment services than, for example, residential treatment or if agencies are more likely, for any other reason, to accept Medicaid insurance for outpatient versus residential services, for example.

Response:

Thank you for raising this interesting point about subsectors. We have now added a different disaggregation approach that relies on treatment agency setting. Unlike the effects on the aggregate outcome, we found a negative association between job postings in residential treatment services and Medicaid expansion. Thus, we are unable to reliably conclude an effect in either direction from this analysis.

5. While the authors hint at increases in OUD treatment use following Medicaid expansions as responsible for changes in SUDT job ads, I think a better argument should be made on the mechanism that leads to changes in job postings after Medicaid expansions.

Response

This is a good suggestion. We revised the discussion section as we did not detect significant increases in online job postings after Medicaid expansion. Our discussion now includes more information about possible mechanisms behind our hypothesized effects.

6. I'm not sure I understand the reasoning behind choosing an OLS model versus a model such as -nbreg- to model count data.

Response:

Our preferred estimation approach is the ordinary least squares (OLS) and log transformation of job postings per residents (our main outcome variable, which is thus not a count measure) as this approach is one of the most widely used and flexible estimation methods. Unlike the OLS models, the Negative Binomial model is appropriate to fit count outcomes. Thus, we have now provided an additional table to compare the estimates using OLS and Negative Binomial models, using a version of our dependent variable that we turn into a count measure. The results are highly similar.

7. Also, why not use a Probit or logit model for dichotomous outcomes?

Response

In the revised version, we manually cleaned the data further following the personal communications with our data partners to include a large number of job postings related to SUDT hospitals, which were previously omitted when using the 6-digit NAICS level. Due to this improved data definition, we observed that every state had at least a job posting every year. Therefore, we do not estimate the effects of Medicaid expansion on the extensive margin of hiring attempts (whether a state had a job posting in a certain year).

8. An event study (ES) is conducted to assess the parallel trends assumption. However, such a model used with more dummies for the post-expansion period (2+ years, 3+ years) is useful at uncovering model dynamics. It is possible that the effect occurs only 1-2 years post expansion or only lasts 1-2 years. In that case, the results as they are, where only one post-expansion dummy is used, are mudded.

Response

Thank you for this suggestion. In the revised version, the event study now uses 4+ years for the post-expansion period.

9. I would also try to lag the Medicaid expansion variable to allow for the effect to be felt by agencies and to allow for the need for additional workforce to develop.

Response:

We have added Table S1 (Appendix). This event study also allows us to observe dynamic estimates. We have added the following text:

“We also conducted a DD analysis with one-year-lagged Medicaid expansion status as it may take time to develop increased workforce size (Model 4, Table S1). We observed no significant difference in the estimates compared to the baseline results.”

10. Finally, in the second part of the two part model, the sample should be restricted to state/year observations with positive numbers of job postings.

Response:

We improved the data set construction and observed that every state had at least one job posting every year. Therefore, we do not estimate the effects of Medicaid expansion on the extensive margin of hiring attempts (whether a state had a job posting in a certain year).
