# Supplementary material for: Job postings in the substance use disorder treatment related sector during the first five years of Medicaid expansion
Source: PLoS One. 2020 Jan 30;15(1):e0228394. doi: 10.1371/journal.pone.0228394 (PMC6992002; doi:10.1371/journal.pone.0228394)
Supplement: Attachment — Submitted filename: Referee Report.pdf [file pone.0228394.s008.pdf]

Referee Report for "Hiring in the substance use disorder treatment related sector during the first five years of Medicaid expansion

Summary:

This paper evaluates whether the Medicaid expansions under the Affordable Care Act (ACA) led to changes in hiring in sectors related to substance-abuse treatment. The authors collect hiring data at the state and year level using data from Burning Glass Technologies which measures online job postings. The authors do not find that Medicaid expansions led to changes in the aggregate number of employment advertising postings for substance-abuse treatment employment in the aggregate, but they do find results for different sub-categories.

Overall, this paper is very clearly written, and given the current focus on opioid-related deaths, answers an important policy question. I have comments and questions for the authors below.

Questions:

1. One of my major questions for the authors concerns the Burning Glass Technologies data. Does the data measure the number of workers firms are seeking to hire or the number of job postings? For example, if I run a SUDT facility and I place one job advertisement seeking to hire three counselors, will that show up as ONE in the BGT data or THREE?

2. I also wondered if the authors' results change if they do not control for the opioid prescribing rates and drug poisoning mortality rates. It seems that both of these rates are plausibly affected by the Medicaid expansions and also by employment in the SUDT areas, so including them in the models might lead to "overcontrolling" bias.

3. I was intrigued by the occupation-specific results, and I have some thoughts on these models. First, the authors state on pg. 9 that, "although (perhaps unsuprisingly given the number of different specifications we run), there are some coefficients that are statistically significant for specific occupations."

   Can the authors adjust their p-values for multiple-hypothesis tests? That might be an easy way to confront whether the large number of hypotheses they test are driving some to be statistically significant by chance.

   The authors talk some in the Discussion (on pg. 10) about the different levels of employment in the SUDT industry. It might be nice to have a slightly longer discussion about the potential mechanisms behind why hiring changed in some SUDT occupations but not in others.

   Finally, I wasn't sure what to make of the occupation-specific results in Figure 4 compared to the all professionals results. The estimate for every single occupation (in both the extensive and overall results) seems to be higher than the all professions results. I would have thought that the all professions results would have been closer to the average of the different occupations. Do the authors have an idea on what's going on? I apologize if I am missing something obvious.

4. I’m confused why the authors didn’t run a Poisson QML model or Negative Binomial model with state and year fixed effects if their data is right-skewed. My understanding is that both of these models allow two way fixed effects, and including robust standard errors in a Poisson model will account for overdispersion.

5. Are there any treatment states that are driving the authors’ results? Can the authors run a “leave out” analysis where the drop one treatment state at a time to see if any states are extremely influential in the aggregate results.
